# Venomous snakes elicit stronger fear than nonvenomous ones: Psychophysiological response to snake images

**DOI:** 10.1371/journal.pone.0236999

**Published:** 2020-08-19

**Authors:** Eva Landová, Šárka Peléšková, Kristýna Sedláčková, Markéta Janovcová, Jakub Polák, Silvie Rádlová, Barbora Vobrubová, Daniel Frynta

**Affiliations:** 1 National Institute of Mental Health, Klecany, Czech Republic; 2 Department of Zoology, Faculty of Science, Charles University, Prague, Czech Republic; 3 Department of Psychology, Faculty of Arts, Charles University, Prague, Czech Republic; University of Wuerzburg, GERMANY

## Abstract

Snakes have been important ambush predators of both primates and human hunter-gatherers throughout their co-evolutionary history. Viperid snakes in particular are responsible for most fatal venomous snakebites worldwide and thus represent a strong selective pressure. They elicit intense fear in humans and are easily recognizable thanks to their distinctive morphotype. In this study, we measured skin resistance (SR) and heart rate (HR) in human subjects exposed to snake pictures eliciting either high fear (10 venomous viperid species) or disgust (10 nonvenomous fossorial species). Venomous snakes subjectively evaluated as frightening trigger a stronger physiological response (higher SR amplitude) than repulsive non-venomous snakes. However, stimuli presented in a block (more intense stimulation) do not trigger a stronger emotional response compared to sequentially presented stimuli (less intense stimulation). There are significant interindividual differences as subjects with high fear of snakes confronted with images of viperid snakes show stronger, longer-lasting, and more frequent changes in SR and higher HR compared to low-fear subjects. Thus, we show that humans demonstrate a remarkable ability to discriminate between dangerous viperids and harmless fossorial snakes, which is also reflected in distinct autonomous body responses.

## 1. Introduction

### 1.1. Snakes as evolutionary threat

Ever since their appearance, primates and early hunter-gatherers have been subject to life-threatening risks from snakes. As a consequence, primates including contemporary humans developed improved visual abilities and superior pre-attentive attention specifically for detecting snakes and other stimuli representing an evolutionary threat [1–4]. Although this predation pressure has left no trace in the fossil record, some circumstantial evidence is available. In an attempt to assess the hazards that snakes pose to primates, McGrew [5] observed a group of chimpanzees in Senegal. Within four years, as many as 142 encounters of snakes belonging to 14 species were recorded. Headland and Greene [6] showed that local populations in some parts of the world have been regularly exposed to predatory attacks by giant constrictor snakes in the recent past. Over the course of four decades, a quarter of Agta Negritos men, a tribe of hunter-gatherers from the Philippines, were attacked by the reticulated python (*Malayopython reticulatus*), resulting in six fatalities [6].

Even today, snakebite envenoming remains a significant health concern. In total, 3 709 snake species are currently being recognized and around 35% of them use venom to kill their prey [7]. In fact, the number of reptile species capable of producing toxins in their saliva may be up as high as 2 000 [8]. Out of these, 250 are listed by the World Health Organization as being medically important [9], especially members of the Elapidae and Viperidae families that possess a very potent venom delivery system [8]. Every year, 4.5–5.4 million people are bitten by snakes worldwide and the estimated death toll ranges from 81,000 to 138,000 [9]. Another 400,000 victims suffer major disabilities such as amputations [9]. Therefore, snakebites have been recently claimed the world’s biggest and grossly underestimated hidden health crisis [10].

### 1.2. Risk of envenoming in different regions

The risk of snake envenomation is the highest in South and Southeast Asia and Sub-Saharan Africa [11, 12]. Southeast Asia is inhabited by several deadly venomous viperid and elapid snakes, e.g., the Russell’s viper (*Daboia russelii*), saw-scaled viper (*Echis carinatus*), Indian cobra (*Naja naja*), monocled cobra (*Naja kaouthia*), and common krait (*Bungarus caeruleus*). Africa is home to some of the most dangerous snakes, e.g., the West African carpet viper (*Echis ocellatus*), Roman's saw-scaled viper (*E*. *leucogaster*), and puff adder (*Bitis arietans*) from Viperidae and the forest cobra (*Naja melanoleuca*), black-necked spitting cobra (*N*. *nigricollis*), and mambas (*Dendroaspis* spp.) from Elapidae. The majority of fatal snakebites in Europe and the Middle East is caused by the Levant viper (*Macrovipera lebetina*), coastal viper (*Montivipera xantina*), and Palestine viper (*Daboia palaestinae*). Rattle snakes (Crotalinae, Viperidae) are the most dangerous snakes in North America, mainly the western diamondback rattlesnake (*Crotalus atrox*) and eastern diamondback rattlesnake (*C*. *adamanteus*). Several viperid snakes pose a significant threat also in South America, e.g., the South American rattlesnake (*C*. *terrificus*), common lancehead (*Bothrops atrox*), jararaca (*B*. *jararaca*), jararacussu (*B*. *jararacussu*), and South American bushmaster (*Lachesis muta)*, as well as deadly venomous elapids, the coral snakes (*Micrurus* spp.) [12, 13]. Viperids are absent from Australia, while many elapids occur there, with the most venomous being the brown snakes (*Pseudonaja* spp.), tiger snake (*Notechis scutatus*), taipans (*Oxyuranus* spp.), and death adders (*Acanthophis* spp.) [13, 14].

To summarize, snakes of the Viperidae family in particular present a major health risk for humans over much of the world except Australia. Consequently, vipers elicit significant fear response and therefore can be used as a salient evolutionarily relevant stimulus in emotion research [15].

### 1.3. Fear module

It has been hypothesized that because of the risk presented by venomous snakes, human ancestors have evolved a complex adaptive system of interconnected fear responses manifested on the psychological, behavioral, physiological, and neural level [16]. This system, according to some authors, has been encapsulated in a specific brain structure, the so-called module of fear localized in the amygdala [16–18] (but see Rosen and Donely [19] who failed to observe amygdala activation in rodents experiencing unconditioned fear). Many years of extensive research have demonstrated that the fear module is particularly triggered by snakes. In contrast to other animals, snakes are associated with a fearful human voice already in infants as young as 9 months [20]. Snakes also capture pre-attentive attention, so they can be spotted much faster than, for example, flowers or mushrooms on a background of distractors [2]. And finally, the psychophysiological fear response elicited by snakes compared to other animate objects is stronger, longer-lasting [21, 22], and can be triggered even unconsciously [23–25, cf. 26].

### 1.4. Variable snake appearance may trigger distinct emotions of fear and disgust

So far, research has been treating snakes as a uniform stimulus category supposedly activating the evolved fear module [27], although different snake taxa are likely to elicit different levels of fear. Although venomous snakes show great pattern and morphological variation, only certain morphotypes are perceived as dangerous and highly fear-evoking, specifically snakes of the Viperidae family (*Crotalinae*, *Viperinae*, and *Azemiopinae*). It has been shown that large body size, conspicuous scales with contrasting patterns, and bright coloration contribute to fear perception [15]. This is congruent with results of a cross-cultural comparison of human fear responses to various venomous and nonvenomous snake species commonly occurring in Europe, Middle East, and North Africa. Both Czech and Azerbaijani respondents rated various species of vipers as the most fear-eliciting. These snakes have characteristic features such as a thick short body, wide distinct neck, and prominent eyes. Interestingly, the Egyptian cobra (*Naja haje*), which is a slender-bodied elapid, was evaluated by the respondents from both countries as the most fear-eliciting and dangerous only when presented in a threatening (in contrast to resting) position [28], highlighting the importance of context (and possibly movement) in fear perception.

Appearance and dangerousness to humans is even more variable across the whole suborder of snakes, including the non-venomous ones. Consequently, some snakes may be perceived as not frightening but highly disgusting [15, 29, 30]. Mainly harmless subterranean (fossorial) snakes from the group of blind snakes called Typhlopoidea (*Xenotyphlopidae*, *Typhlopidae*, *Leptotyphlopidae*, *Gerrhopilidae*, and *Anomalepididae*) are less fear-evoking, but highly repulsive [15]. These findings raise an intriguing question: do snake species advertise the danger they present to humans, for example, their toxicity, through their appearance?

From the functional perspective, fear and disgust are biologically adaptive and genetically fixed intense responses to potentially life-threatening situations [31]. Although both negative emotions should lead to avoidance/withdrawal [32], they can be clearly distinguished on the physiological, psychological, and behavioral level [33, 34]. While fear is elicited by the presence of a predator (e.g., a snake) or other imminent threat posing a direct risk of physical harm or even death [35–37], disgust has originally developed as a food-rejection emotion. Its main function is to prevent the transmission of illness or disease through ingestion of contaminated objects [38, 39]. Thus, it triggers disease-avoidance behavior [40, 41] as a part of the “behavioral immune system” [42]. However, understanding of the psychophysiological differences between emotions in general is still insufficient [43, 44], particularly between fear and disgust. The fear response involves activation of the sympathetic nervous system, which initiates the “fight or flight” reaction characterized by heart rate acceleration [45–47]. Disgusting stimuli, on the other hand, have highly variable physiological effects on heart and respiratory rates [44]. Skin conductance, which is determined by the activity of sympathetically innervated sweat glands, is reported to increase with both fear and disgust [44, 48].

However, some authors have challenged the view of basic emotions as biologically fixed, universally shared, discrete entities that serve a specific function in survival each having a distinct facial expression, physiological pattern and neural substrate. For example, Russell [49] conceptualized emotions as simple affective states called the core affects, which can be either good or bad and energized or enervated and were attributed to some internal or external cause. Similarly, Barrett [50] highlighted that despite people’s belief of being able to recognize their own emotions, research has not yet identified clear criteria for the presence of a certain emotion. Therefore, according to her model, an emotional experience arises when affective feeling is cognitively categorized based on our knowledge.

### 1.5. Autonomic electrodermal and heart rate response to snakes

Since the 1970s, an extensive series of experiments has demonstrated that snakes, compared with other stimuli, selectively trigger a stronger and longer-lasting physiological fear response, particularly an increase in heart rate, blood pressure, and skin conductance, which is more resistant to extinction [22]. Its main purpose is to mobilize energy reserves and prepare the body for rapid action [cf. 51]. The majority of studies did not measure a spontaneous response to snakes but rather used a within-subject controlled differential conditioning paradigm in which some fear-relevant (snakes and spiders) and fear-irrelevant (flowers and mushrooms) stimuli were followed by an electric shock (CS+), while others were not paired with any shocks (CS-). Most often, the dependent variable was the skin conductance response (SCR), alternatively also heart rate (HR). It was argued that the difference in SCR to the CS+ vs CS- stimulus should be bigger for fear-relevant than for fear-irrelevant stimuli, which was then supported by several studies [23, 25, 52–55], for a review see [16, 21]. For example, Soares and Öhman [54] reported that both fearful and non-fearful control subjects had significantly larger differential electrodermal responses to pictures of snakes and spiders than to pictures of flowers and mushrooms. It was also shown that SCR triggered by fear-relevant compared with fear-irrelevant stimuli was more resistant to extinction, however, no effect of fear-relevance on HR was found [53]. Interestingly, a backwardly masked presentation (stimulus appears on the screen for only about 30 ms) or an instruction that no shock will follow may wipe out differential SCR to neutral stimuli, but has no effect on differential SCR to fear-relevant stimuli [23, 54, 55]. Öhman and Soares [25] demonstrated on SCR changes that subjects can be non-consciously conditioned by electric shocks to fear snakes and spiders but not flowers and mushrooms even when these are masked during acquisition.

Others have used a different approach and instead of conditioning normal subjects to fear snakes, they directly measured spontaneous physiological responses of respondents with low vs high (phobic) fear of snakes. It has been repeatedly shown that high-fear individuals display larger SCR and increased HR when exposed to snakes compared to control stimuli [56] and, moreover, their SCR in response to snakes is elevated compared to low-fear individuals under both conscious and unconscious (masked) presentation conditions [24].

In regards to autonomous fear responses to snakes in particular, there is an ongoing debate in the literature whether these are acquired through direct aversive experiences (or observation of others’ behavior) during development [57], as the studies using classical Pavlovian fear conditioning might suggest, or snake fear is rather biologically prepared (genetically fixed), universal trait in humans and other animal species that can be manifested even without any prior negative experience (humans: [20], birds: [58], geckos: [59], primates: [60, 61]).

### 1.6. Study aims

Even though there is a substantial body of literature dealing with psychophysiological responses to snakes (live or on pictures), those studies fail to reflect the immense morphological and pattern variety of different snake taxa that can trigger various emotions. Often researchers use the snake as a uniform stimulus prototypically eliciting fear and no attention is being paid to characteristics of that particular species (in a majority of studies the species is not even specified) such as its body size, color pattern, posture, toxicity, etc. However, from our previous research, we know that this is crucial as it can significantly affect our responses [15, 28]. Moreover, as mentioned above, most of earlier studies measured physiological parameters using a differential conditioning paradigm, which is a qualitatively different phenomenon than spontaneous reactions of unconditioned individuals and therefore, such an approach is not ideal for studying traits that might be biologically prepared.

Viperid snakes are unique stimuli for humans in several aspects: 1) many species of viperid snakes currently pose a serious risk of venomous snakebite on all the continents except Australia and Antarctica [13] and thus exert an important selective pressure on the human ability to perceive, emotionally evaluate, and avoid these snakes; and 2) it was demonstrated cross-culturally that humans perceive the specific viperid morphotype (including the families *Crotalinae*, *Viperinae*, and *Azemiopinae)* as highly fear-eliciting [15, 28]. Thus, viperid snakes present an ideal model group for studying the effect of fear response when spotting a snake.

In this study, we focused on psychophysiological responses of human subjects elicited by 20 species of snakes belonging to two distinct groups differing in their morphology, ecology, behavior, toxicity (dangerousness), and fear/disgust-evoking properties. Although autonomous bodily responses (mostly skin conductance and heart rate) to snakes have been already well-explored in previous research, to our knowledge, no one has ever focused on interstimulus variability within the category of snakes. There is evidence that people distinguish between different snake species emotionally by experiencing either fear or disgust. It is thus reasonable to expect that the same distinction is reflected in physiological responses as well. For the first time, this would show that snakes are an emotionally ambivalent category, which might have significant implications for future research by highlighting the importance of careful stimulus selection. Moreover, it would also be beneficial to the clinical practice as it might tailor treatment of snake phobics to their specific needs of better emotional regulation. In our previous study, we demonstrated a difference in fear and disgust evaluation of various snake species between people with low and high fear of snakes and disgust propensity [62]. Thus, it would be interesting to see whether these interindividual differences also manifest in physiological parameters. Finally, comparing two distinct categories of snakes as stimuli might reveal an adaptive pattern of specific physiological response targeted to venomous viperid snakes.

First, we aimed to examine the autonomous physiological response to venomous viperid snakes eliciting high fear but low disgust compared to non-venomous fossorial snakes eliciting low fear but high disgust. This exploratory question has never been studied, although based on the research using other fear- and disgust-eliciting animal stimuli, one might assume that while fear-eliciting snakes should trigger the sympathetic (predatory defense) nervous system activating the body energy resources and leading to increased skin conductance and heart rate, disgust-eliciting worm-like snakes should activate the parasympathetic (behavioral immune) nervous system causing increased skin conductance but decreased heart rate. Second, we will study how physiological responses might be affected by different levels of snake fear and disgust propensity. Again, although a few studies have compared physiological responses of snake phobics versus healthy controls, no one has incorporated disgust into the model, which might shed more light on psychopathological dynamics of snake phobia development.

Previous psychophysiological research on snake fear has used various methodology which makes any comparisons difficult. Therefore, we have chosen to study the differences in physiological responses; first, between stimulus categories and then between subjects using two experimental designs, i.e., sequential (presentation of an individual picture stimulus followed by an interstimulus) and block presentation (presentation of 10 pictures one right after the other with no interstimulus in-between) that might provoke an emotional response of different intensity. Specifically, we assume that stimulation in a block design should trigger a more intense physiological response compared to stimulation in a sequential design. Finally, as the current literature is not consistent regarding correspondence between self-reported emotions and physiological parameters, we will investigate the link between evaluation of snake stimuli and physiological responses.

## 2. Materials and methods

### 2.1. Participants

We recruited respondents with different levels of fear of snakes and general disgust propensity as measured by commonly used psychometric questionnaires–the Snake Questionnaire (SNAQ [63], in a Czech translation [64]) and the Disgust Scale-Revised (DS-R [65, 66], in a Czech translation [67]). The respondents were selected so that the dataset would be balanced with comparable numbers of respondents with high and low scores from each of the above-mentioned questionnaires. The high-fear/disgust participants were defined as those scoring above the upper quartile on the SNAQ/DS-R scales (upper quartiles were computed for Czech population: SNAQ score ≥ 8 [64]; DS-R score ≥ 52 [67]). The respondents also completed the Emotion Reactivity Scale (ERS [68]) and provided information about their gender, age, and field of study. In total, 161 individuals were included in the study. Out of these, 143 respondents performed the sequential design experiment (139 of them completed all the questionnaires; 75 high-fear, 64 low-fear, 59 high-disgust, 80 low-disgust; 116 females, 27 males; 46 biological education, 97 non-biological education; mean age 28.12 ± 10.65) and 143 the block design experiment (all of them completed the questionnaires; 82 high-fear, 61 low-fear; 59 high-disgust, 86 low-disgust; 118 females, 25 males; 46 biological education, 97 non-biological education; mean age 28.0 ± 10.18). Both experimental designs were performed by 125 respondents with the second experiment being carried out after at least a month-long period, so that the effect of habituation would be minimized. The sample size was based on both, previous studies (for the comparison, see Table 4) and a statistical a priori power analysis computed in G*Power 3 [69]. This analysis was conducted to test the difference in physiological responses to three categories of stimuli (fear/disgust/control, see below) between two main categories of respondents (high/low fear of snakes) using an ANOVA, a medium effect size (f = 0.25) and an alpha of 0.05. The result showed that a total sample of 158 participants in one experimental design was required to achieve a power of 0.80. However, the prevalence of people with high fear of snakes who are willing to attend an experiment with snakes is rather low, thus, we compromised on having 143 respondents in each design. The sample is not perfectly balanced especially in terms of gender; however, our main aim was to keep the ratio of respondents with high and low fear of snakes balanced. As the prevalence of snake fear is higher in women [64, 70], our study included more women than men.

### 2.2. Stimuli

**Fig 1:**
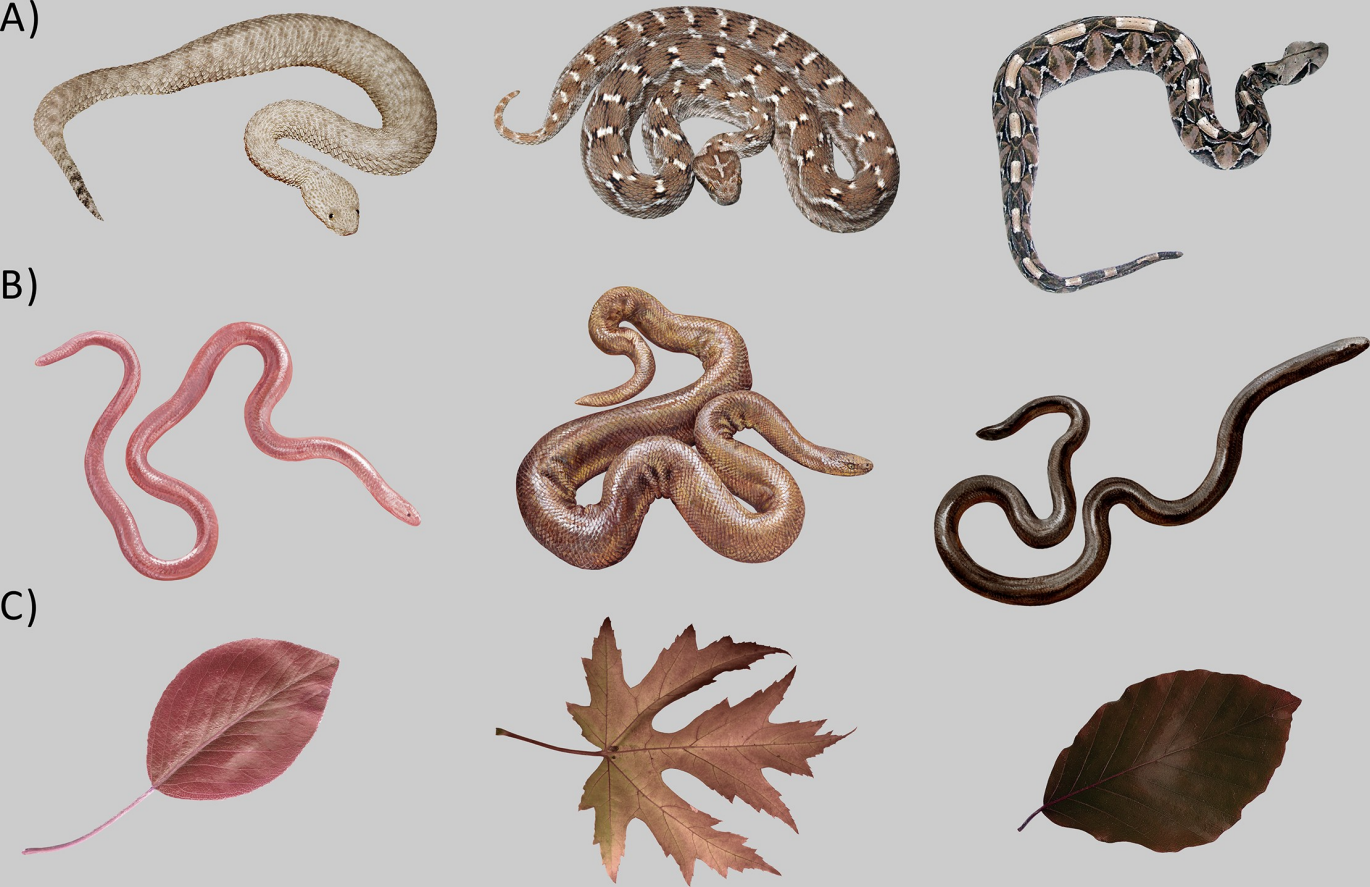
Illustrative examples of picture stimuli used in the study. Due to copyright restrictions, some of the pictures have been replaced by different photos/illustrations that were not used in the study but are essentially very similar. The snake species are as follows, from left to right: A) fear-eliciting snakes: Sahara sand viper (*Cerastes vipera*), photo by Milan Kaftan, Sochurek's saw-scaled viper (*Echis carinatus sochureki*), photo by Tomáš Mazuch, and Gaboon viper (*Bitis gabonica*), photo by Milan Kaftan; (B) disgust-eliciting snakes: Eurasian blind snake (*Xerotyphlops vermicularis*), northern rubber boa (*Charina bottae*), and brahminy blindsnake (*Indotyphlops braminus*), all three illustrations have been painted by Pavel Procházka (please note that due to copyright restrictions, the photos used in the study had to be replaced by illustrations for the purpose of this illustrative figure only); (C) control stimuli (leaves): silver birch (*Betula pendula*), photo by Silvie Rádlová, Old World sycamore (*Platanus orientalis*), photo by Petra Frýdlová, and European beech (*Fagus sylvatica*), photo by Eva Landová.

We selected 20 photographs of snake species evoking a strong and distinct emotional response based on their morphotype according to the self-reported evaluation (15)– 10 dangerous (highly venomous) viperid snakes evoking strong fear (for their venom characteristics, see Table 1) and 10 disgust-eliciting harmless (nonvenomous) fossorial snakes, evoking only a weak fear response (see also Fig). On a 7-point Likert scale of fear (1 = no fear, 7 = extreme fear), the fear-eliciting snakes scored high (mean score 5.15 ± 1.95), while the disgust-eliciting ones scored much lower (mean score 3.24 ± 2.00) [62]. As emotionally neutral controls, we used 20 photographs of tree leaves (see also Fig 1 for examples of the tested species in each category depicting their morphological variety). According to a preliminary study with 135 respondents, leaves do not elicit any fear (mean score 1.09 ± 0.53), nor disgust (mean score 1.09 ± 0.54; 7-point scale, 1 = no fear/disgust). The photos used in the study were either taken by the authors themselves or downloaded from the Internet; in this case they were licensed under the Creative Commons and/or a written permission for scientific use was obtained from their authors. For the full list of included snake species, see S1 Table. The photos were standardized by placing the stimuli on a unified grey background and resized to assume a similar relative size in a 2:3 ratio of the picture.

**Table 1:** List of fear-eliciting species included in the study and their venom and physiological parameters.

Latin name	English name	Subfamily	LD50 (IV)	Length (mm)	Venom (mg)	Danger	Venom ratio	Sources
*Atheris squamigera*	Variable bush viper	Viperinae	0.611	800	2.5	3.0	0.0031	[71–73]
*Azemiops feae*	Fea’s viper	Azemiopinae	0.52	800	1.75	1.5	0.0022	[71, 74, 75]
*Bitis gabonica*	Gaboon viper	Viperinae	0.55	1800	750	5.5	0.4167	[71, 75, 76]
*Bitis nasicornis*	Rhinoceros viper	Viperinae	0.55	2000	848	5.5	0.4240	[71, 77, 78]
*Cerastes vipera*	Sahara sand viper	Viperinae	0.5	490	43	2.5	0.0878	[71, 79]
*Crotalus adamanteus*	Eastern diamondback rattlesnake	Crotalinae	1.33	2440	500	4.5	0.2049	[71, 75, 80]
*Echis carinatus multisquamata*	Multiscale saw-scaled viper	Viperinae	3.26	625	25	4.5	0.0400	[75, 81, 82]
*Echis carinatus sochureki*	Sochurek’s saw-scaled viper	Viperinae	2.98	625	25	4.5	0.0400	[75, 81, 82]
*Protobothrops jerdonii*	Jerdon’s pitviper	Crotalinae	1.5	990	300	3.0	0.3030	[71, 75, 83]
*Vipera orlovi*	Orlov’s Viper	Viperinae	0.608	500	4	2.5	0.0080	[71, 77]

LD50 (IV) = 50% lethal dose (intravenous), the amount of venom injected intravenously, which kills 50% of mice, it is the measure of venom toxicity; Danger = the index of dangerousness to humans as retrieved from Clinical Toxinology Resources (toxinology.com: University of Adelaide, Australia) ranging from 0 = not at all dangerous till 6 = extremely dangerous; Venom ratio = the ratio of venom amount to body length.

To analyze the effect of morphologic characteristics of the examined snake species on the human responses, we included the following measures and color characteristics with considerable variability within the two snake categories (fear- and disgust-eliciting) as explanatory variables: body length, neck width, eye size, proportion of white, black, grey, red, brown, and blue colors, mean saturation and standard deviation of saturation (for more information on the measurement and extraction of these variables, see Rádlová et al. [15]. We also included three venom characteristics of the fear-eliciting snakes: LD50 (50% lethal dose, intravenous), index of dangerousness as retrieved from the Clinical Toxinology Resources [75], and a ratio of venom volume to body length (Table 1).

### 2.3. Procedure and apparatus

To fulfill the aims, we examined several skin resistance (SR) and heart rate (HR) parameters (see Table 2) in response to images of fear-eliciting venomous viperids, disgust-eliciting non-venomous fossorial snakes, and leaves as control stimuli. We also adopted two experimental designs to examine different intensities of visual stimulation. In the first one, further referred to as the sequential design, the pictures of snakes and leaves were presented individually in an alternating order starting with a control stimulus (i.e. leaf–venomous snake–leaf–disgusting snake and so on repeated through the entire presentation of 40 images), each presented for 5 seconds and separated with a black screen (interstimulus) presented for 5 seconds or until the participant calmed down, whichever lasted longer.

**Table 2:** Overview of the used variables. Unless otherwise stated, the values were computed separately for each stimulus category.

Abbreviation	Variable	Definition
**Skin resistance (SR)**		
NR	Number of reactions	Total number of reactions
MAS	Mean amplitude per stimulus	The sum of all the amplitudes divided by the number of stimuli
MDS	Mean duration of reaction per stimulus	The sum of all the reaction durations divided by the number of stimuli
MAR	Mean amplitude per reaction	The sum of all the amplitudes divided by the number of reactions
MDR	Mean duration per reaction	The sum of all the reaction durations divided by the number of reactions
**Heart rate (HR)**		
HR slope	Heart rate slope	Slope of linear regression of heart rate in time

**Fig 2:**
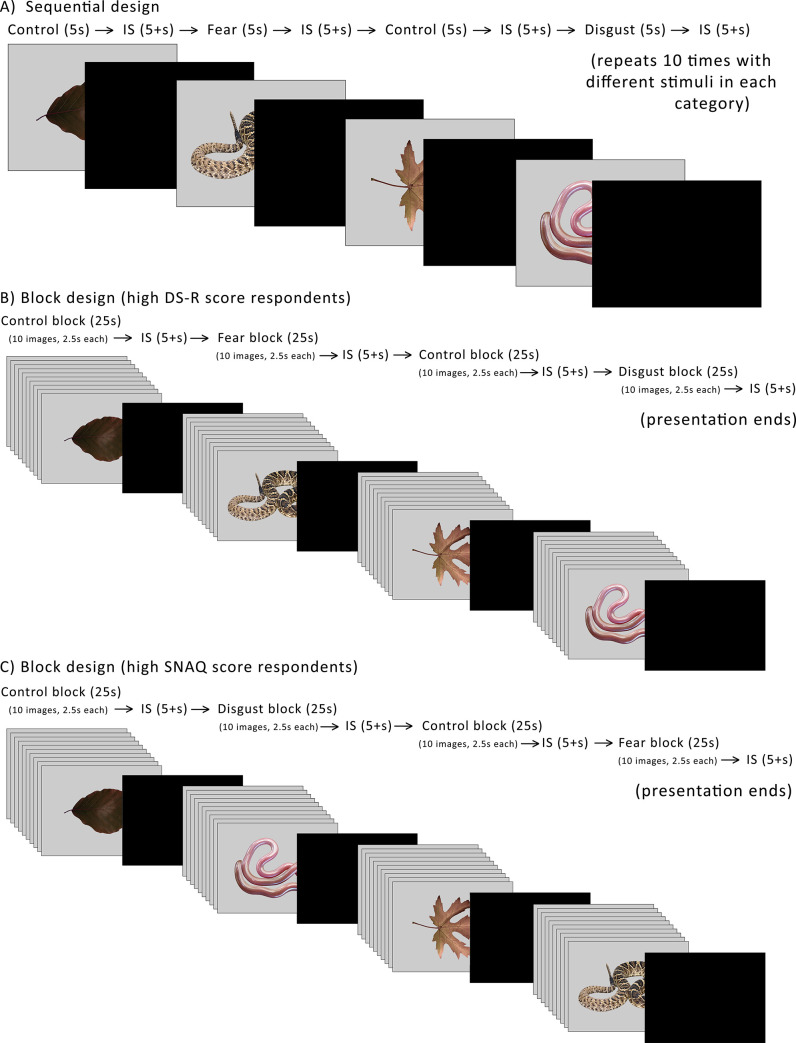
Visual diagram of the experimental design. The presentation always started with the control stimuli. A) Sequential design–the pictures of fear-eliciting snakes (fear), disgust eliciting snakes (disgust), and leaves (control) were presented on a computer screen individually in an alternating order, each presented for five seconds and separated with a black screen (interstimulus, IS). The interstimuli were presented for five seconds or until the participant calmed down, whichever took more time. The presented scheme was repeated ten times, each time with a different picture from the same category. B, C) Block design–similar to the sequential design, but the stimuli were presented in blocks of ten pictures not interlaced with the IS, which only followed after the presentation of all 10 pictures of the block. Each block consisted of ten different pictures from the same category, i.e. the same stimuli as used in the sequential design. The two block designs differed in the order in which the fear and disgust stimuli were presented: participants with high disgust propensity scores were presented the (B) order, while participants with high snake fear were presented the (C) order.

In the second experimental design, further referred to as the block design, the pictures were presented in blocks consisting of 10 pictures from a single category (fear/disgust/control). This design, commonly used in fMRI and EEG studies, is hypothesized to present stronger stimulation compared to individually presented stimuli (also called event-related design in fMRI/EEG studies). We applied it to physiological measurement to compare the effect of these two designs of visual stimulation on the physiological response and we plan to compare the results of the block design with a subsequent fMRI experiment. The pictures in the block design were presented one right after the other (with no black screen in-between) and each picture from the specific category appeared on the screen for 2.5 seconds only, i.e. the entire block was shown for 25 seconds. This was followed by a black screen presented for at least 5 seconds or more if necessary, for the respondent to calm down (see Fig 2).

Snake illustrations in this preview have been made by Pavel Procházka, photos of leaves taken by Petra Frýdlová and Eva Landová. Please note that during the experiment, photos of real snakes were used.

High-fear participants were presented with the fear-evoking block at the end of the presentation, and similarly, high-disgust participants viewed the disgust-evoking block as the last one. This was mainly due to methodological reasons to ensure that the physiological response we were most interested in would not be compromised by object novelty. Additionally, this design was also more suitable for the high-fear/disgust subjects as they were exposed to the strongest stimuli at the end of the trial. Low-fear and low-disgust participants viewed these two presentations in a random order (in total, 70 respondents started with the fear-evoking snakes and 73 with the disgust-evoking ones). Respondents, who attended both experiments (n = 125) did so in a random, counter-balanced order.

Moreover, 111 respondents from the main sample (59 high-fear, 52 low-fear, 49 high-disgust, 62 low-disgust, 93 females, 18 males, 40 with biological education, mean age 27.89 ± 8.41) rated all depicted snake species for fear and disgust. We adopted a well-established method used in a number of previous studies [84–86]. The photographs of snakes (360 x 540 pixels) were presented one by one on a computer screen in a random order. The respondent was asked to score fear or disgust elicited by each species on a 7-point Likert scale (1 –not disgusting/fear-evoking at all, 7 –the most disgusting/fear-evoking) in two separate tasks, the first scored emotion was chosen randomly. The rating was performed after the main experiment to minimize the effect of habituation.

For measuring physiological responses, we used Multifunction Biotelemetry Support System for Psychophysiology Monitoring VLV3 [87], which enables measuring and evaluating multiple physiological variables in real time during the stimuli presentation. Skin resistance (SR) was measured using dry sensors attached to the second phalanx of the index and middle fingers of the non-dominant hand. Heart rate (HR) was measured with a pair of standard Ag/AgCl electrodes attached by adhesive collars to the skin under the right collarbone and the left fifth intercostals. To analyze the reactions, we measured length (from the beginning of the SR change curve to the peak of the curve) and amplitude of the SR change curve, which corresponds to the intensity of the emotional reaction. The heart activity was recorded as mean HR (in beats per minute) in the given time period. The pictures (1772 x 1181 pixels, 300 DPI resolution) were presented on a computer screen (26", 2560 x 1440 resolution, full screen presentation) placed 55 cm from the edge of the table. The respondents were asked to leave their hands with attached sensors on the table and to watch the screen during the whole presentation without unnecessary movements.

This study was carried out in accordance with the approval of the Ethical Committee of the National Institute of Mental Health no. 55/16, with the written informed consent from all subjects in accordance with the Declaration of Helsinki.

### 2.4. Statistical analysis

For the variables used to characterize physiological responses, see Table 1. They were used as raw data when possible and were transformed for use of linear models, using either logarithmic or square root transformations to approximate to the normal distribution. The distribution of model residuals was visually inspected for both deviations from normality and variance heterogeneity. The Spearman’s correlation coefficient was computed to compare the self-reported evaluation and physiological responses. To test the differences in physiological responses to individual snake species and to different stimulus categories, we performed a Friedman test and post hoc Nemenyi test as implemented in the R package PMCMR [88]. A Mann-Whitney U test was used to compare the physiological reactions of high- and low- fear/disgust respondents. The above-mentioned tests were used as a non-parametric alternative for raw data deviating from normality, as we aimed to maintain extreme values of highly fearful participants. Two analyses were used to examine the contribution of respondent’s characteristics (gender, age, education, SNAQ, DS-R, and ERS scores) to the physiological responses; these were used as explanatory variables in linear mixed effects models (LME; implemented in R package nlme), which allowed for inclusion of the effects of respondent’s characteristics accounted for the individual identity using it as a random factor. An ANOVA was applied to test the effect of explanatory variables. We also performed an exploratory redundancy analysis (RDA; implemented in the R package vegan [89]), which is a multivariate direct gradient method. It extracts and summarizes the variation in a set of response variables (parameters of physiological reactions) that can be explained by a set of explanatory variables. This analysis permits to plot both response and explanatory variables to a space defined by the extracted gradients and enables detection of redundancy (i.e., shared variability) between sets of response and explanatory variables. Statistical significance of the gradients was confirmed by permutation tests. Repeatability was computed as another exploratory analysis to test the intra-individual consistency between respondents performing both tasks using the R package rptR [90]. Repeatability allowed us to establish the relative contribution of between-individual variation to the overall variation [91, 92]. Calculations were performed in R [93] and Statistica [94].

## 3. Results

### 3.1. Sequential design of individual stimuli presentation

#### 3.1.1. Differences in the physiological response to fear- and disgust-eliciting snakes

**Fig 3:**
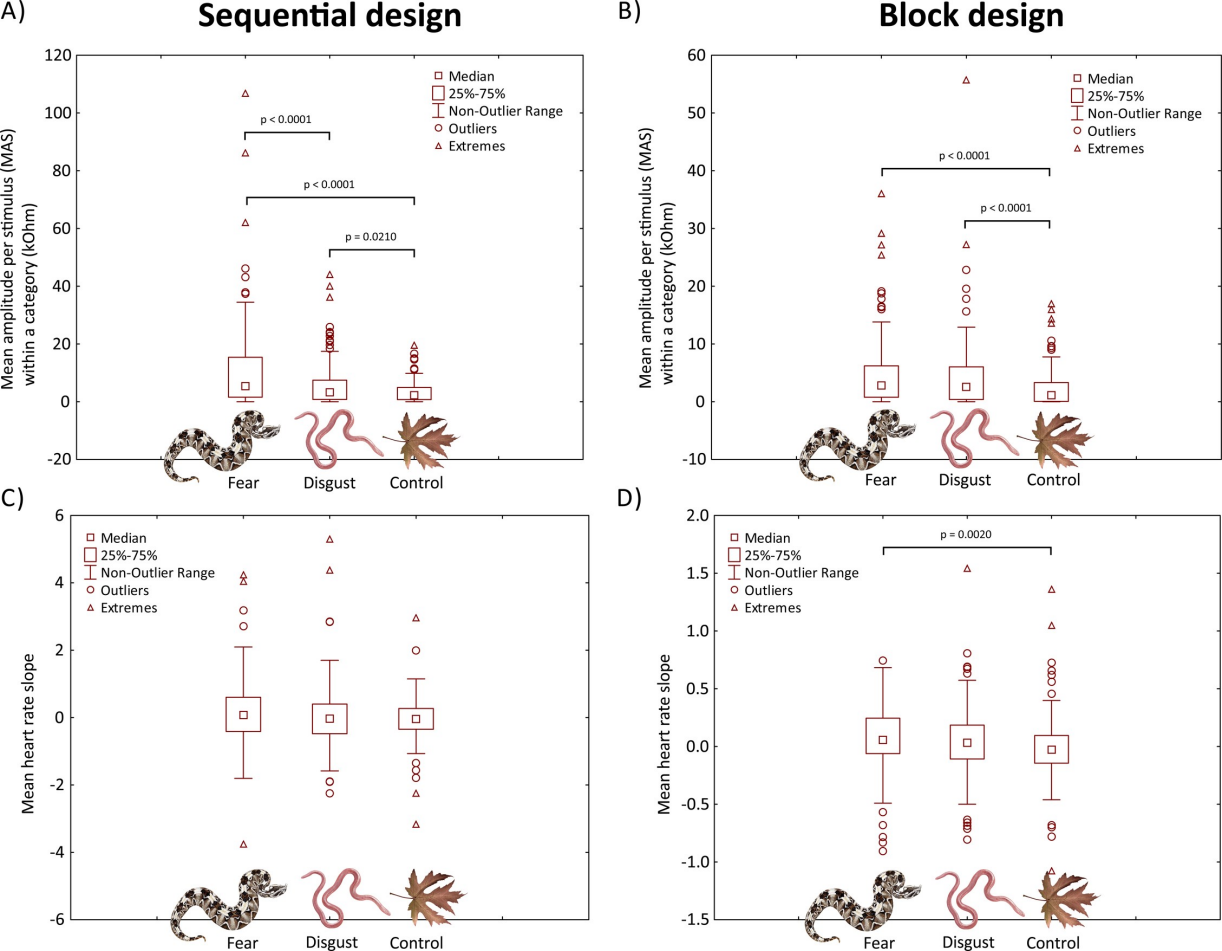
Boxplots of differences between fear, disgust, and control conditions in measurements of (a, b) galvanic skin resistance (SR) and (c, d) heart rate (HR). Significance of the difference was measured using the Friedman and post-hoc Nemenyi tests. Depicted species: rhinoceros viper (*Bitis nasicornis*) as a fear-eliciting snake, Eurasian blind snake (*Xerotyphlops vermicularis*) as a disgust-eliciting snake (both illustrations by Pavel Procházka), and Old World sycamore (*Platanus orientalis*) as a control stimulus, photo by Petra Frýdlová.

We pooled the data for individual stimuli and performed further analyses with the mean SR parameters for each stimulus category (fear-eliciting snakes, disgust-eliciting snakes, and leaves as control stimuli). To fully compare the physiological responses to a given category, we computed the number of reactions (NR), mean amplitude/duration of reaction per stimulus (MAS and MDS, respectively; i.e. the sum of the amplitudes/durations divided by the number of stimuli in each category), and mean amplitude/duration per actual reaction (MAR and MDR, respectively; i.e., the sum of the amplitudes/durations divided by the number of reactions in each category). NR describes the frequency of any SR reaction regardless its intensity or overall respondent’s responsiveness, MAR and MDR describe the quality of the response (e.g. intensity) and MAS and MDS combine both quantity and quality in one parameter. We included the respondents with no skin resistance reaction (n = 9), too, because this represents a relevant result for people with no fear of snakes. Mean number of reactions was 14.04 ± 9.37. We also computed the mean HR slope for each stimulus category (fear/disgust/control), i.e., the slope of the linear regression line through the data points, which describes the change in heart activity in time. It was computed for the five-second interval of each stimulus presentation and subsequently as a mean for all stimuli in a given category. Positive slope values indicate an increase in HR in time, while negative values indicate a decrease, and zero slope indicates no change (for overview of the variables, see Table 1).The Friedman test revealed that the effect of category on all tested SR parameters was highly significant (Friedman NR χ^2^ = 74.711, MAS χ^2^ = 58.682, MDS χ^2^ = 74.652, MAR χ^2^ = 27.106, MDR χ^2^ = 25.879, all df = 2, all p < 0.0001; for the visualization, see Fig 3). Furthermore, we performed a pairwise comparison of the stimulus categories using the post-hoc Nemenyi test. All of the comparisons were significant (p values from 0.0210 to < 0.0001) except for MAR and MDR, where the disgust vs control comparison was not significant (p > 0.05). Therefore, the SR responses to fear-eliciting snakes are significantly differentiated from those to the control stimuli in all examined parameters. However, when responses to disgust-eliciting snakes are compared to controls, the differences lie rather in the response frequency rather than in their amplitude or length.

For the HR slope, the result of the Friedman test was not significant, however, the visualization shows there is a slight tendency for higher HR in response to fear-eliciting snakes and lower HR in response to disgust-eliciting snakes compared to controls (Fig 3). When we performed the analysis separately for high-fear respondents, the result was marginally significant (Friedman χ^2^ = 5.5254, df = 2, p-value = 0.0631). The subsequent post-hoc Nemenyi test was also marginally significant for the comparison of fear-evoking snakes and controls (p = 0.056) and not significant in other cases (for more analyses of high-fear respondents, see below).

#### 3.1.2. Effect of respondents’ fear of snakes and disgust propensity

Next, we analyzed the effect of respondents’ scores on the Snake Questionnaire (SNAQ, a measure of snake fear), Disgust Scale-Revised (DS-R, a disgust propensity measure), and Emotion Reactivity Scale (ERS, a measure of emotional sensitivity, intensity, and persistence) and other characteristics, e.g., the gender, age, biological or non-biological education, stimulus category, and interactions of the respondent’s characteristics with the stimulus category on the SR response. We used an LME model that allows to include the effects of respondent’s characteristics accounted for individual identity.

Regarding the amplitude (MAS), five explanatory variables remained in the final reduced model: category, DS-R, age, ERS and ERS*category interaction. The ANOVA revealed that only the effects of stimulus category (F_2,274_ = 42.7670, p < 0.0001) and ERS*category interaction (F_2,274_ = 8.4200, p = 0.0003) were significant. In the case of duration (MDS), seven explanatory variables remained in the final reduced model: stimulus category, SNAQ, DS-R, ERS, gender, SNAQ*category and ERS*category interactions. The ANOVA revealed that the effects of category (F_2,272_ = 58.5345, p < 0.0001), SNAQ (F_1,134_ = 7.6997, p = 0.0063), SNAQ*category interaction (F_2,272_ = 9.9585, p = 0.0001) and ERS*category interaction (F_2,272_ = 5.4564, p = 0.0047) were significant.

Furthermore, we employed an RDA with the same explanatory variables except the stimulus category. The analysis generated three constrained axes that explained 9.3% of the full variability. The sequential "Type I" ANOVA (n permutations = 20 000) revealed that the effect of SNAQ scores (F_1,135_ = 5.2362, p = 0.0097), ERS (F_1,135_ = 3.7564, p = 0.0344), and age (F_1,135_ = 4.8459, p = 0.0191) on the mean physiological parameters (MAS, MDS, and NR) were significant. Thus, the examined individual characteristics have a significant effect on the SR response, however, they explain only a small portion of the full variability. On the other hand, the effect of stimulus category on the HR slope was not significant. Five explanatory variables remained in the final reduced model: stimulus category, SNAQ, gender, age and SNAQ*category interaction. The ANOVA revealed that only the effects of SNAQ (F_1,111_ = 17.4560, p = 0.0001), age (F_1,111_ = 7.2836, p = 0.0080) and SNAQ*category interaction (F_2,226_ = 6.6595, p = 0.0015) remained significant.

**Fig 4:**
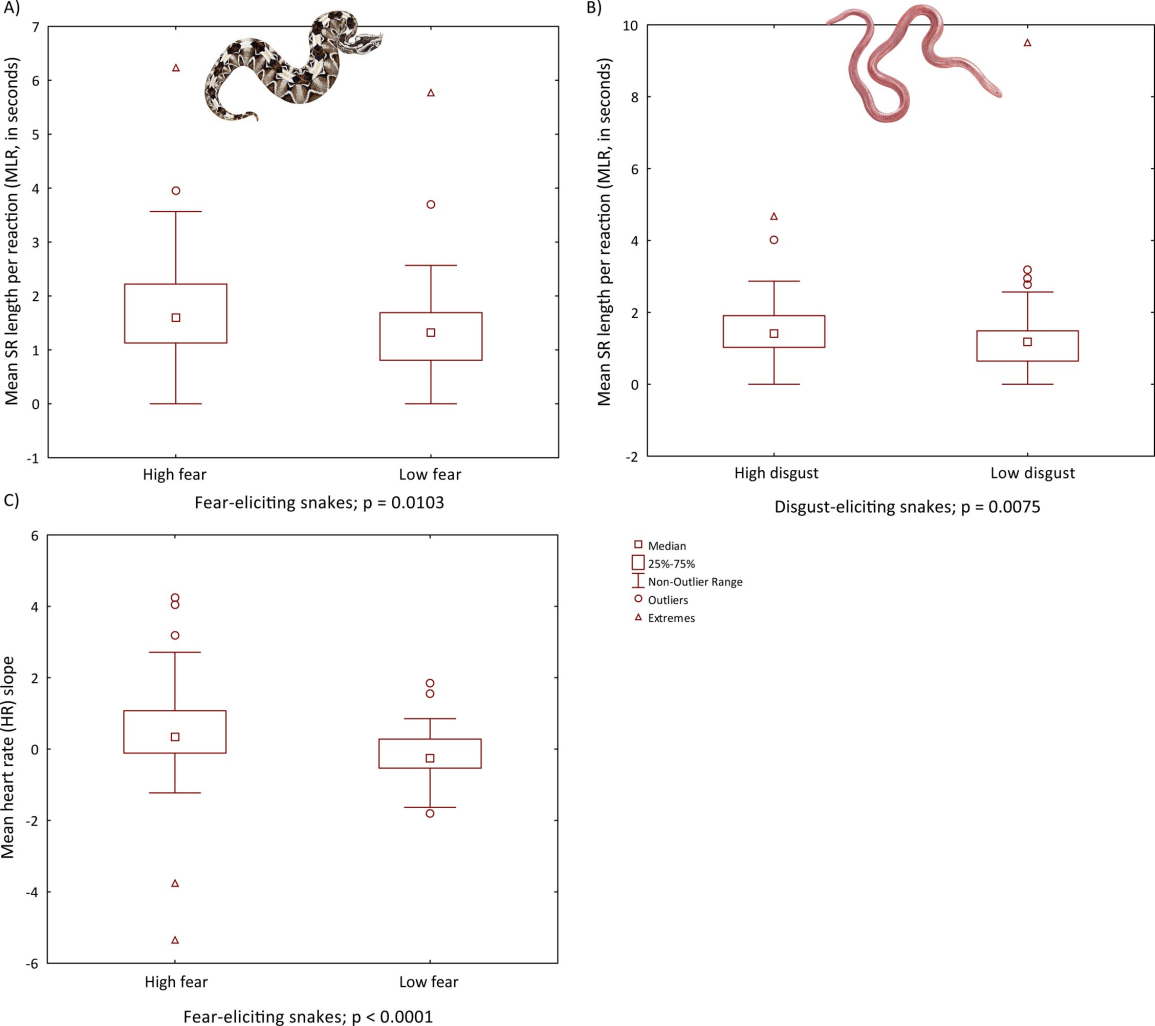
Boxplots of differences in (a, b) skin resistance (SR) and (c) heart rate (HR) between respondents with high snake fear or disgust propensity and controls in the sequential design. Significance of the differences was measured using the Mann-Whitney U test. Depicted species: rhinoceros viper (*Bitis nasicornis*) as a fear-eliciting snake and Eurasian blind snake (*Xerotyphlops vermicularis*) as a disgust-eliciting snake. Illustrations by Pavel Procházka.

We then analyzed differences in the examined SR parameters between high- and low-fear and high- and low-disgust respondents using the Mann-Whitney U tests. As for high- and low-fear respondents, the comparisons were significant in the case of number and duration of reactions to fear-eliciting snakes (NR p = 0.0187, MDS p = 0.0129 and MDR p = 0.0103; Fig 4), but nonsignificant in the case of amplitude and reactions to other categories of stimuli. As for high- and low-disgust respondents, the comparisons were significant for all the examined parameters in responses to both the fear- and disgust-eliciting snakes (for fear-eliciting snakes all p < 0.05, for disgust-eliciting snakes all p < 0.01; Fig 4), except for MDR, which was significant only for disgust-eliciting snakes (p < 0.01). Thus, respondents differing in the disgust propensity level demonstrate not only different reactions to disgust- but also fear-eliciting snakes. Furthermore, the Mann-Whitney U test revealed a significant difference in the HR slope for fear-evoking snakes when comparing low- vs high-fear respondents (p < 0.0001), but no significant difference when comparing high- vs low-disgust respondents.

### 3.2. Block design of stimuli presentation

#### 3.2.1. Differences in the physiological response to fear- and disgust-eliciting snakes

To compare the physiological response not only between the blocks of stimuli of discrete categories (fear/disgust/control), but also between both designs (intra-individual consistency, see below in section 3.3.), we computed the same mean variables for both designs: NR, MAS, MDS, MAR, MDR, and HR slope for the whole block of each stimulus category (see Table 1). The mean number of reactions was 12.29 ± 7.77 and respondents with no skin resistance reaction were included (n = 7). Similarly to the sequential design, the Friedman test revealed a highly significant effect of stimulus category on all the measured SR parameters (Friedman NR χ^2^ = 24.852, MAS χ^2^ = 43.758, MDS χ^2^ = 40.950, MAR χ^2^ = 22.535, MDR χ^2^ = 17.112, all df = 2, all p < 0.0001, except for MDR: p = 0.0002; for the visualization, see Fig 3). However, based on the post-hoc Nemenyi test, both snake categories significantly differed from controls (p values ranged from 0.0030 to < 0.0001), but the difference in the SR response to fear- and disgust-eliciting snakes in the block design was not significant in any of the comparisons. Thus, compared to the sequential design, there is always a significant difference between disgust and control stimuli, but not between fear and disgust stimuli. The result of the Friedman test was also significant for the HR slope (Friedman chi-squared = 11.529, df = 2, p-value = 0.0031). However, unlike for SR, the only significant difference in the HR slope was between the fear-eliciting snakes and controls as revealed by the post-hoc Nemenyi test (p = 0.002).

#### 3.2.2. Effect of respondent’s fear of snakes and disgust propensity

Subsequently, we analyzed the effect of respondent’s individual characteristics (SNAQ, DS-R, and ERS score, gender, age, and biological vs non-biological education), the stimulus category, and their interactions on the SR response using the LME models. The results (see below) supported the crucial effect of stimulus category on the SR changes. In the case of amplitude (MAS), five explanatory variables remained in the final reduced model: category, SNAQ, gender, age, and SNAQ*category interaction. The ANOVA subsequently revealed that only the effect of stimulus category (F_2,282_ = 22.3322, p < 0.0001), SNAQ (F_1,139_ = 8.6516, p = 0.0038), and SNAQ*category interaction (F_2,282_ = 5.7753, p = 0.0035) were significant. In case of duration (MDS), five explanatory variables remained in the final reduced model, four of them were significant: category (ANOVA, F_2,280_ = 23.2492, p < 0.0001), SNAQ (F_1,140_ = 12.1516, p = 0.0007), SNAQ*category (F_2,280_ = 5.1285, p = 0.0065), and DS-R*category interaction (F_2,280_ = 4.6166, p = 0.0107), while the effect of DS-R was not significant.

**Fig 5:**
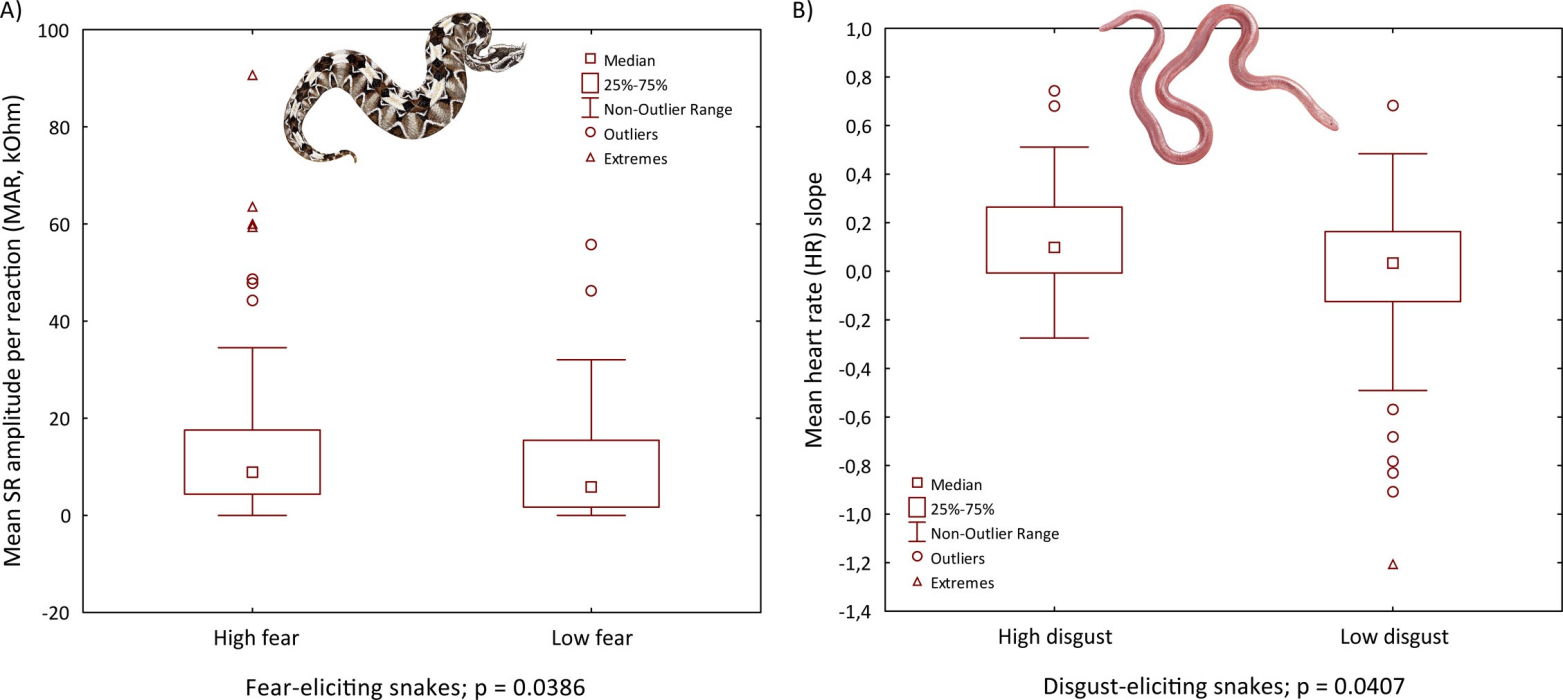
Boxplots of differences in (a) skin resistance (SR) and (b) heart rate (HR) between sensitive respondents and controls in the block design. Significance of the differences was measured using the Mann-Whitney U test. Depicted species: rhinoceros viper (*Bitis nasicornis*) as a fear-eliciting snake and Eurasian blind snake (*Xerotyphlops vermicularis*) as a disgust-eliciting snake. Illustrations by Pavel Procházka.

We also performed an RDA, which generated 3 constrained axes that explained 12.6% of the full variability. The sequential "Type I" ANOVA (n permutations = 20 000) revealed a significant effect of SNAQ scores (F_1,103_ = 6.7917, p = 0.0013), age (F_1,103_ = 3.1672, p = 0.0407), and gender (F_1,103_ = 4.8827, p = 0.0115) on the mean SR parameters (NR, MAS, and MDS). However, we did not find a significant effect of stimulus category on the HR slope. Seven explanatory variables remained in the final reduced model: category, DS-R, education, age, ERS, age*category interaction, and ERS*category interaction. The ANOVA revealed that only the effect of age*category interaction was significant (F_2,266_ = 5.6566, p = 0.0039). Thus, in both experimental designs, the effect of stimulus category on the HR change was not significant. We also performed Mann-Whitney U tests to analyze the differences in SR responses between pre-defined groups of respondents with high and low fear of snakes and disgust propensity. As for high- and low-fear respondents, the comparisons were significant for MDS (p = 0.0322) and MAR (p = 0.0386) in response to fear-eliciting snakes, as well as for all the parameters in response to disgust-eliciting snakes (p values from 0.0419 to 0.0022; Fig 5).

Thus, in the block design, different levels of fear of snakes affected more the reactions to disgust- but not fear-eliciting snakes. Unlike in the sequential design, there were no significant differences between respondents with high and low disgust propensity in the block design. Furthermore, the Mann-Whitney U test revealed no significant difference in the HR slope when comparing low- and high-fear respondents. For the comparison of high- vs low-disgust respondents, the only significant difference was in the reactions to fear-evoking snakes (p = 0.0407).

### 3.3. Comparison of the two designs

#### 3.3.1. Effect of experimental design

From the above-presented results, we concluded that the effect of experimental design was not negligible and examined it further employing LME models. The experimental design and respondent’s characteristics (SNAQ, DS-R, and ERS scores, gender, age, and biological or non-biological education) were used as explanatory variables. The ANOVA revealed that in the case of MAS for all stimuli, the effects of design (F_1,124 =_ 9.0677, p = 0.0032), SNAQ (F_1,118_ = 5.7467, p = 0.0181), and age (F_1,118_ = 4.2450, p = 0.0416) were significant. For MDS, the effects of design (F_1,124_ = 7.1368, p = 0.0086) and SNAQ (F_1,118_ = 10.9151, p = 0.0013) were significant. And for MDR, only the effect of SNAQ (F_1,118_ = 8.3484, p = 0.0046) was significant. The results were comparable when computed for mean reactions to all stimuli and to fear- and disgust-eliciting snakes separately. However, in the case of MAR, the results were different when computed for all stimuli and both snake categories separately. For all stimuli, the effects of gender (F_1,118_ = 6.7606, p = 0.0105) and age (F_1,118_ = 5.7223, p = 0.0183) were significant, in the case of fear-eliciting snakes the effects of design (F_1,124_ = 12.2090, p = 0.0007), gender (F_1,118_ = 4.5649, p = 0.0347), and age (F_1,118_ = 5.3629, p = 0.0223) were significant. Conversely, there was no significant effect on the reactions to disgust-eliciting snakes. Thus, the experimental design affects especially the mean reactions per stimulus, which corresponds to the reaction frequency rather than its amplitude or duration and is higher in the sequential design. The amplitude of the SR reaction (MAR) is higher in the sequential design only in response to fear-eliciting snakes.

#### 3.3.2. Intra-individual consistency

We moreover examined intra-individual consistency of SR responses in respondents who performed both experimental designs (n = 125) by computing repeatability. The results were highly significant for NR (R values from 0.392 for control stimuli to 0.563 for all stimuli, all p < 0.0001), MAS (R values from 0.384 for disgust-eliciting stimuli to 0.454 for all stimuli), and MDS (R values from 0.351 for disgust-eliciting stimuli to 0.491 for all stimuli), except for MAS in response to fear-eliciting stimuli, which was significant at p = 0.0003 (R = 0.301). As for MAR and MDR, the results were highly significant in the case of MDR in response to control stimuli (R = 0.337, p < 0.0001) and significant at the p level from 0.0067 to 0.0004 in all other cases (MAR: R values from 0.222 for disgust-eliciting to 0.3 for control stimuli; MDR: R values from 0.224 for disgust-eliciting to 0.241 for fear-eliciting stimuli). We then computed repeatability for high-fear respondents (n = 70). Overall, the results were significant except for MDR in response to disgust-eliciting and control stimuli. For the control stimuli, the repeatability R level was lower in all parameters and higher in the case of amplitude in response to snake stimuli and all stimuli. Thus, despite a significant effect of the design, the SR responses were relatively highly intra-individually consistent across both designs in most examined cases. For the complete repeatability results, see Table 3.

**Table 3:** Results of repeatability of physiological responses.

		All respondents (incl. HF)			HF respondents only		
		R	CI	p	R	CI	p
mean amplitude per stimulus	**all**	0.454	0.299, 0.585	< 0.0001	0.516	0.334, 0.667	< 0.0001
	**fear**	0.301	0.128, 0.450	0.0003	0.313	0.099, 0.502	0.0043
	**disgust**	0.384	0.216, 0.518	< 0.0001	0.481	0.283, 0.637	< 0.0001
	**control**	0.401	0.251, 0.545	< 0.0001	0.29	0.072, 0.496	0.0077
mean duration of reaction per stimulus	**all**	0.491	0.349, 0.607	< 0.0001	0.465	0.263, 0.631	< 0.0001
	**fear**	0.360	0.197, 0.500	< 0.0001	0.306	0.074, 0.509	0.0051
	**Disgust**	0.351	0.194, 0.493	< 0.0001	0.365	0.152, 0.550	0.0009
	**control**	0.443	0.301, 0.574	< 0.0001	0.336	0.109, 0.516	0.0023
mean amplitude per reaction	**all**	0.249	0.082, 0.403	0.0026	0.342	0.118, 0.530	0.0019
	**fear**	0.260	0.088, 0.418	0.0018	0.368	0.146, 0.581	0.0009
	**disgust**	0.222	0.043, 0.394	0.0068	0.325	0.101, 0.517	0.0031
	**control**	0.300	0.129, 0.452	0.0004	0.25	0.029, 0.464	0.0194
mean duration per reaction	**all**	0.226	0.061, 0.385	0.0060	0.232	0.010, 0.432	0.0279
	**fear**	0.241	0.083, 0.401	0.0036	0.242	0.016, 0.440	0.0231
	**disgust**	0.224	0.054, 0.388	0.0063	0.178	0.000, 0.388	*0*.*0752*
	**control**	0.337	0.167, 0.480	0.0001	0.176	0.000, 0.395	*0*.*0767*
number of reactions	**all**	0.563	0.425, 0.686	< 0.0001	0.539	0.313, 0.689	< 0.0001
	**fear**	0.471	0.291, 0.599	< 0.0001	0.422	0.179, 0.598	0.0007
	**disgust**	0.508	0.325, 0.629	< 0.0001	0.507	0.270, 0.671	< 0.0001
	**control**	0.392	0.196, 0.521	0.0001	0.296	0.019, 0.487	0.0145

HF = high fear, R = repeatability, CI = confidence interval; all p values were significant except for two (MDR disgust and control in HF respondents), which are highlighted in italics.

### 3.4. Correlation of self-reported emotions and physiological response

**Fig 6:**
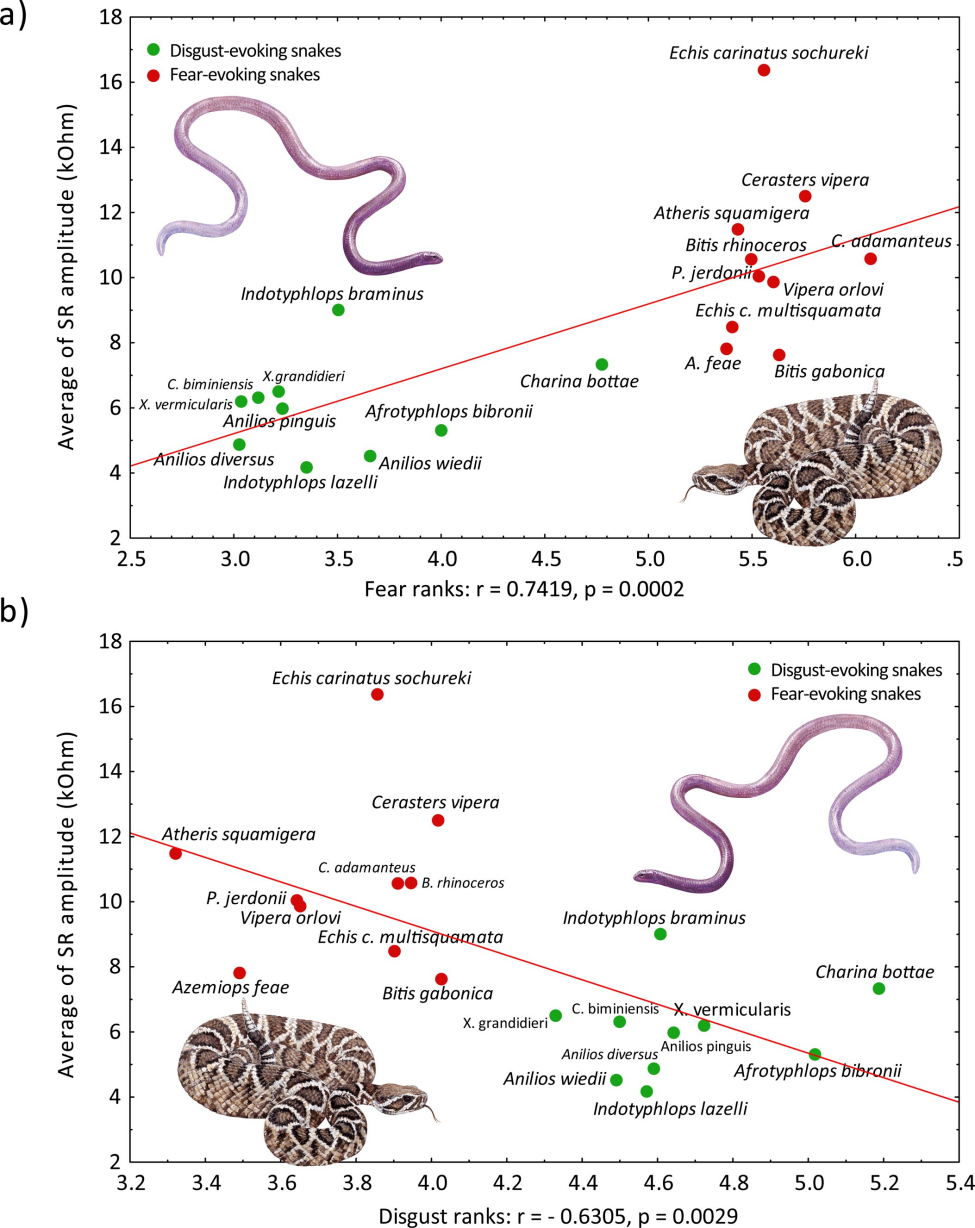
Spearman’s correlation between physiological responses and scores of the snake stimuli (sequential design). Both (A) fear scores and (B) disgust scores of fear- and disgust-eliciting snake stimuli closely correlated with the average of skin resistance amplitude. The venomous Viperid snakes (the variable bush viper *Atheris squamigera*, Fea’s viper *Azemiops feae*, Gaboon viper *Bitis gabonica*, rhinoceros viper *Bitis nasicornis*, Sahara sand viper *Cerastes vipera*, eastern diamondback rattlesnake *Crotalus adamanteus*, multiscale saw-scaled viper *Echis carinatus mutlisquamata*, Sochurek’s saw-scaled viper *Echis carinatus sochureki*, Jerdon’s pitviper *Protobothrops jerdonii*, and the Orlov’s viper *Vipera orlovi*) were scored as more fear-eliciting (and less disgust-eliciting) and also elicited stronger physiological reactions than harmless fossorial snakes (the Bibron’s blind snake *Afrotyphlops bibronii*, northern blind snake *Anilios diversus*, rotund blind snake *Austrotyphlops pinguis*, brown-snouted blind snake *Anilios wiedii*, Bahamian slender blind snake *Cubatyphlops biminiensis*, northern rubber boa *Charina bottae*, brahminy blind snake *Indotyphlops braminus*, Hong Kong blind snake *Indotyphlops lazelli*, Madagascar blind snake *Xenotyphlops grandidieri*, and the Eurasian blind snake *Xerotyphlops vermicularis*). Depicted species: eastern diamondback rattlesnake as a fear-eliciting snake and Madagascar blind snake as a disgust-eliciting snake. Illustrations by Pavel Procházka.

In the current study, we measured the physiological response to 10 venomous fear-eliciting snakes and 10 harmless disgust-eliciting snakes (see S1 Table for more details on the snake species in both categories). To examine the relationship between the self-reported evaluation and physiological response, we computed Spearman’s correlations. Mean fear or disgust score of each snake species reported by the respondents was highly correlated with the mean SR amplitude of the response to the respective snake (fear: Spearman’s r = 0.7729, p = 0.0001; disgust: Spearman’s r = - 0.6827, p = 0.0009; Fig 6).

### 3.5. Testing homogeneity of physiological responses within categories of fear- and disgust-eliciting snakes—exploratory analysis

As most analyses were based on a comparison of means of distinct stimulus categories, we furthermore explored the homogeneity within the categories of snake stimuli defined by Rádlová et al. [15], whether the SR responses correspond to the same distinct categories of two types of snakes. The Friedman test showed that the differences in the SR amplitude between snakes within the pre-defined categories were significant (fear: Friedman χ2 = 73.02, df = 9, p < 0.0001; disgust: Friedman χ2 = 45.435, df = 9, p < 0.0001). However, the post-hoc Nemenyi test revealed that only one species from each snake category significantly differed from the others in the mean SR amplitudes: in the case of fear-eliciting snakes, the Sochurek's saw-scaled viper differed from all the other snake species except the Sahara sand viper (*Cerastes vipera*), thus, only 8 out of 45 comparisons were significant (p < 0.05). In the case of disgust-eliciting snakes, the brahminy blind snake (*Indotyphlops braminus*) differed from the other snake species except the northern blind snake (*Anilios diversus*), Eurasian blind snake (*Xerotyphlops vermicularis*), rotund blind snake (*Anilios pinguis*), and the northern rubber boa (*Charina botae*), thus, only 5 out of 45 comparisons were significant (p < 0.05). The results calculated for SR duration were in this case almost identical to those for the amplitude and therefore will not be further mentioned in the text.

The Friedman test comparing the responses to individual species was significant. Therefore, we used a redundancy analysis (RDA) to further examine the contribution of nine morphological and three venom characteristics (Table 2) of relatively more diverse fear-eliciting snakes (treated as explanatory variables—constraints) to the SR amplitude. However, the model showed no constrained component. Thus, these analyses supported the hypothesis that the selected snake species present a homogenous category based on both the self-reported evaluation and physiological measures, regardless of their morphological or venom variability.

## 4. Discussion

In the current research using two experimental designs, we directly compared autonomous physiological responses of human subjects exposed to snake pictures of two distinct emotional and zoological categories. One composed of viperid snakes that are all venomous, dangerous to humans and evoke intense fear, the other one including fossorial snakes that are non-venomous, harmless, and evoke mainly disgust and repulsion. We have demonstrated that the fear-eliciting venomous snakes trigger a significantly more pronounced physiological response as evidenced by higher SR amplitude compared with the disgust-eliciting snakes, while no significant difference could be found in HR. Furthermore, we provide evidence that the individual level of snake fear greatly effects bodily responses as high-fear subjects show more increased response in both SR and HR parameters compared with low-fear subjects.

Although people demonstrate a measurable emotional response in both the SR and HR channels upon seeing a snake picture, measuring SR was a much more sensitive and robust method in the current study. By analyzing the curve of SR changes, we can reliably discriminate between reactions to fear-eliciting viperids and disgust-eliciting fossorial snakes. In other studies, the measurement of electrodermal activity was sensitive enough to detect differences in reactions to phobia-triggering animal stimuli (snakes and spiders) compared to controls even in a masked condition, when the stimuli were presented only for 30 ms [23, 24]. Fredrikson and Öhman [53] in their detailed study of fear conditioning likewise measured both parameters, i.e., the SR as well as HR response to snakes and spiders (fear-relevant stimuli). They found that electrodermal responses conditioned to fear-relevant stimuli, once learned, showed a resistance to extinction compared to neutral stimuli. However, this was not the case for HR, nor did they find differences in HR during acquisition or habituation phases, in contrast to SR. Similarly, Bradley, Cuthbert, & Lang [95] showed that HR after the stimulus onset first increased (1 s), then decreased (2–3 s), and then increased again (4–5 s), but no such pattern was detected for SR. This phasic HR response is also typical for anticipated threat situations [96]. For all these reasons, HR as a psychophysiological response parameter may be highly dependent not only on intensity of the stimulus, but also the details of a particular experimental design, e.g., the time for which the stimulus is presented to the subject or whether the subject has a chance to somehow predict when the stimulus is going to appear.

It is noteworthy that the way of visual emotional stimulation plays a significant role [95, 97–99]. It can be demonstrated that stimuli presented sequentially elicit a different level of emotional response compared to a stronger visual stimulation by 10 consecutive stimuli in a block design. When using a single stimulus presentation (sequential design), we can better differentiate responses to fear- and disgust-eliciting snakes and at the same time, the reactions to fear-eliciting are stronger in a sequential than in a block design. The question remains as to why the psychophysiological responses measured in the block design are lower. It can be argued that the block design facilitates habituation and this effect is even more pronounced in the category of fear-eliciting snakes. However, it has been shown that repeated presentation of pictures of similar affective valence does not lead to habituation and the emotional response measured by corrugator electromyographic (EMG) activity is maintained across more than twenty trials [91]. Surprisingly, in our experiment a stronger visual stimulation in the block design did not necessarily lead to a stronger emotional response, particularly if fear-eliciting snakes are shown. This may have considerable implications for fMRI or PET studies where similar picture block designs are commonly used to study the emotional response [100–102].

There have already been numerous psychophysiological studies using snakes as emotionally relevant stimuli. However, not all of them could be easily compared with the present work due to substantial methodological differences (e.g., conditioned electrodermal responses to masked stimuli [23, 25, 52–55], which is not the same as unconditioned spontaneous responses examined here). For a more detailed comparison, we have selected 11 studies meeting at least one of the following criteria: 1) measurement of electrodermal activity or heart rate in response to snakes compared to fear-irrelevant control stimuli or 2) comparison of responses to snakes in respondents with high and low fear of snakes (see Table 4, which compares mean changes in the physiological response between given categories of stimuli or respondents). Most of the studies found higher or more frequent changes in electrodermal activity in response to snake stimuli compared to neutral controls and stronger reactions in snake fearful participants, which is consistent with our results. The results of HR changes were not as robust, however, there was a trend for HR acceleration in reaction to fear-eliciting snakes compared to controls (significant in the block design) and higher HR acceleration in fearful respondents (significant in the sequential design), which is also in agreement with results of the previous studies.

**Table 4:** An overview of results from 11 previous studies compared to ours.

Study	Measured parameters	Participants N	Type of stimuli	HR snakes	EDA snakes	HR snakes HF vs LF	EDA snakes HF vs LF
Courtney et al. 2009 [103]	EDA	32	Pictures, CG pictures, CG videos		↑ HF		
Courtney et al. 2010 [104]	HR, EDA	38	Pictures, CG pictures, CG videos	↑ HF	↑ HF		
Craske & Sipsas 1992 [105]	HR	65	Live			↑	
Dimberg et al. 1998 [56]	HR, EDA	56	Pictures	↑	↑	↑	↑
Flykt & Caldara 2006 [106]	HR	27	Pictures	=		=	
Flykt et al. 2017 [107]	HR, EDA	12	Pictures (un-/masked)	↑/ =	↑/ =		
May 1977 [108]	HR, EDA	24	Pictures, words	↑	↑/ =	↑	↑
Öhman & Soares 1994 [24]	EDA	48	Pictures (un-/masked)		↑		↑
Sánchez-Navarro et al. 2018 [99]	HR, EDA	54	Pictures	↑ HF	↑		
Schaefer et al. 2014 [109]	EDA	42	Videos		↑		=
Wikström et al. 2004 [110]	EDA	51	Words (un-/masked)		↑		=
Present study	HR, EDA	161	Pictures	↑/ =	↑	↑/ =	↑

Only comparable parameters were included in the table: mean heart rate (HR) or electrodermal activity (EDA) change in response to fear-eliciting snakes compared to control stimuli (for all respondents, unless otherwise stated) and a comparison of these two parameters in response to snakes in respondents with high (HF) and low (LF) fear of snakes (↑ higher reaction, = no significant difference, ↑/ = significant in some cases, blank cells indicate that no such analysis was performed in the study). CG = computer generated stimuli.

### 4.1. Effect of individual characteristics on the emotional response to fear- and disgust-evoking snakes

Further intensification of the emotional reaction can be attributed to variable sensitivity of subjects to emotions in general (ERS), their physiological reactivity (repeatability in different parameters of physiological response), and specifically increased snake fear (corresponding to the SNAQ score). Even when filtering out the individual variability in LME models, the intensity and duration of emotional response (SR) is still affected by the subject’s emotional reactivity as measured by the ERS, especially in response towards fear-eliciting snakes. Duration of the SR response is also specifically influenced by the subject’s preexisting snake fear. Individuals experiencing higher levels of snake fear tend to demonstrate longer reactions and increased HR in response to fear-eliciting viperid snake images. These effects apply when we measure reactions to a single stimulus, however, when presented in the block design, there is no measurable effect of emotional reactivity (ERS) either on SR, or on HR. In either case, snake fear still significantly influences the intensity (amplitude) and duration of SR response. As snake fear seems to be a crucial variable affecting a range of measured psychophysiological parameters which is also supported by the literature [56, 63, 102, 111, 112], it is necessary to examine the differences between individuals with low and high snake fear in more detail.

Subjects with high fear of snakes experience more frequent and longer SR reactions (NR, MDS, MDR; see Table 2 for the abbreviations’ explanation) to individually presented viperid snakes, but there is no difference in intensity (amplitude). Snake fearful respondents also show increased HR. In the block design, individuals with high snake fear demonstrated longer SR reactions (MDS) and a higher mean amplitude per reaction (MAR). In the block design, differences in the measured emotional reaction between individuals with low and high snake fear were smaller, but the latter ones demonstrated longer SR reactions (MDS) and a higher mean amplitude per reaction (MAR). In responses to the disgust-evoking fossorial snakes, we only found differences in the block design, where high-fear respondents reacted more strongly in all the parameters of SR (the number of reactions, their amplitude and duration). A similar effect has been observed in snake phobics or participants with a high level of snake fear, where snake pictures provoked an increased number of SR reactions [99] or a larger skin conductance response [56]. These subjects also showed HR acceleration during exposure to snake pictures [56, 99].

When analyzing differences in reactions of individuals with low and high disgust propensity, it seems that the latter ones tend to react more intensely to snakes in general. They show increased SR (the number of reactions, their amplitude and duration) in response not only to the fear-eliciting viperids, but also the repulsive fossorial snakes. It can be argued that people with higher disgust propensity react more strongly to any snake picture irrespective of its morphotype. Individuals with high disgust propensity also show increased HR, but only in response to viperid snakes presented in a block design. However, it is noteworthy that the association between disgust (presumably activating mainly the parasympathetic nervous system) and HR is a complex one often with opposite effects (for example disgust-associated decrease in HR in blood-injection phobia [113]). However, even if these individual characteristics, i.e., snake fear or emotional reactivity, are associated with the measured psychophysiological parameters, the overall explained variability remains as little as 9–12% (based on the RDA). The largest effect is attributable to the stimulus category, i.e., whether the subject is looking at a fear-eliciting viperid or a disgust-eliciting fossorial snake (or alternatively a leaf as a control stimulus).

As the variability of physiological parameters might be caused not only by external factors (in this case the experimental design), but also intrinsic inter-individual differences, we calculated repeatability of the SR parameters to establish the relative contribution of respondent’s individuality to the overall variation (see Table 3). In our results, repeatability was the highest for responses to all the stimuli pooled together irrespective of specific variables, while NR, MAS, and MDS were the most individually repeatable variables. It has been previously shown that HR has an exceptionally good repeatability when measured twice in the same design [114]. In our study, we found relatively high repeatability of SR parameters as well, even across different experimental designs of stimuli presentation (single stimulus vs. block of stimuli). This is fairly surprising given the fact that the mean repeatability of behavioral traits in animals is r = 0.37 [115]; no meta-analysis for humans was found. The fact that there is significant repeatability even across very different experimental designs shows that there are consistent inter-individual differences in SR, which account for 30–50% of the overall variability.

For people with high fear of snakes, the intensive reactions to both snake groups (amplitudes) were more repeatable as opposed to reactions to control stimuli. In general, fear-eliciting stimuli (viperids) evoke more repeatable individual reactions (SR) in many parameters reflecting the intensity of emotional response (MDR, MAR) than fossorial snakes, despite the fact that fear-eliciting snakes often evoke more extreme fear responses.

### 4.2. Psychophysiological response to viperid snakes and how it might affect venom activity

Based on our previous study, all viperids clearly belong to the fear-eliciting group of snakes [15]. Here, we demonstrate that they evoke a fear response of similar intensity on the physiological level too, irrespective of their toxicity or relative threat presented to humans. Here we hypothesize that the observed higher psychophysiological response to viperid snakes is a result of ancestral prioritization in terms of early recognition as well as associated emotion of fear. Furthermore, this autonomous bodily response might be adaptive in a specific interaction with the main components of snake venom. Conversely, it might be argued that the distinct physiological response to viperids is not driven by higher fear, but is merely based on specific low-level visual features that differ between the two studied groups of snakes (i.e., size of scales, head shape, tail shape, body posture, etc.). However, people still report significantly higher fear of vipers. Moreover, some of our subjects demonstrated an increased physiological response to fossorial snakes that do not possess those visual cues. Therefore, it seems that both attention and emotion play a key role. To separate their influence, another experimental design using artificially created rather than natural stimuli would have to be employed.

Besides venom characteristics, there are additional factors contributing to the level of dangerousness of a particular snake species to humans, mainly its body size (which also corresponds to the venom expenditure [116]), level of defensiveness (aggression), as well as the species’ abundance and distribution that influences the probability of encounter (see Table 2). Toxicity of the fear-eliciting snakes from the Viperidae family that we tested is highly variable. The most dangerous snakes for humans are vipers of the genus *Bitis* (*B*. *gabonica a B*. *rhinoceros*) and *Echis* (*E*. *carinatus multisquamata* and *E*. *carinatus sochureki*) with cardiotoxins directly affecting heart activity. Specifically, they cause a decrease in myocardial contractility, as well as disturbances in atrio-ventricular conduction and reduction in amplitude and duration of the action potential [117]. Their venom has a systemic effect on the body, is more toxic and, consequently, causes significantly more fatalities (about 10–20% of envenomings may be fatal). Another very dangerous snake from this subfamily is the eastern diamondback rattlesnake that alongside to the above-mentioned possesses also myotoxins, which can directly affect heart activity (via non-enzymatic destruction of the cardiac muscle [118, 119]). On the other hand, even though the venom of the Sahara sand viper (*Cerastes vipera*) has similar effects (procoagulants, hemorrhagins), its bite does not pose such a high risk of lethality [120]. The Sahara sand viper is a small-sized snake that releases a low amount of venom, which only has a local impact of low efficacy and can resolve even without medical intervention. Similarly, the remaining species (i.e. the Orlov’s viper *Vipera orlovi*, Fea’s viper *Azemiops feae*, and variable bush viper *Atheris squamigera*) are rather smaller snakes producing less venom that predominantly specialize in feeding on amphibians, reptiles, and small-sized mammals [71, 73].

It can be argued that alteration of heart rate activity is a parameter common to both snake venom action and the corresponding psychophysiological emotional response. Fear in general (not only of snakes) operates through activation of the sympathetic nervous system and hypothalamic–pituitary–adrenal (HPA) axis, which leads to a significant increase in heart rate [121]. In the case of disgust evoked by other types of snakes, which is probably mediated by the parasympathetic nervous system, we might expect considerably smaller or even opposite effects [122, 123]. The interaction between venom of viperid snakes and psychophysiological changes might therefore vary depending on the underlying emotion, i.e., fear or disgust.

It has long been known that some snake venoms dramatically lower the blood pressure in human victims and experimental animals [124–126]. This effect could either be caused directly by specific hypotensive agents present in the venom or indirectly through pulmonary vascular obstruction and coronary ischemia [127]. As venom of viperid snakes may affect HR [128], which is also affected by intense fear of snakes, we propose a hypothetical interaction between elicited fear and venom spreading and action following a snakebite. However, it is necessary to distinguish between different timeframes when emotional state of the patient and efficacy of venom may interact.

Immobilization is used as first aid immediately after a snakebite to reduce spreading of venom of viperids [129] and elapids [130]. In this early stage, high fear that increases HR might lead to snake venom spreading faster in the body with negative consequences for the victim’s survival. Experiencing disgust, on the other hand, might have an opposite effect by decreasing HR through parasympathetic activation. However, in later stages, the interaction between fear or disgust, their associated physiological changes, and the venom might depend more specifically on the particular composition of toxins. Interestingly, there are various hypotensive agents in toxins contained in venom of viperid snakes which have been extracted to develop drugs for treating hypertension [128]. When these hypotensive compounds take effect, the blood pressure drastically drops. Therefore, we may hypothesize that the counter-effect of fear increasing the blood pressure might potentially improve the physiological response to envenoming by these snakes. However, no clear-cut prediction for the interaction with disgust can be made, due to its highly variable effect on heart rate. This phenomenon is worth studying.

Alternatively, the stronger physiological response to venomous snakes found in our study might as well be explained by the need of activating energetic resources in dangerous situations, which is necessary for a fast and effective defense (fight-or-flight) response before a snakebite can be delivered, i.e., eliminate the source of threat or rather withdraw oneself from the snake’s presence. However, in their latest review on presumed preparedness of fear of snakes, Coelho et al. [51] argue, that most snakebites happen at very close vicinity and are extremely fast, so the victim usually has no chance to effectively respond.

## 5. Conclusions

The psychophysiological response to images of fear-eliciting venomous snakes from the family Viperidae is higher than the response evoked by images of fossorial, disgust-eliciting snakes. Interestingly, more intensive visual stimulation (i.e., presented longer in a block of ten subsequent images) does not lead to a stronger emotional response than less intensive stimulation (presentation of single images). It would be interesting to explore the effect of different modes of visual stimulation (e.g., comparing the effect of pictures, videos, and live snakes) on the emotional response of human subjects to fear-eliciting snakes.

Our study showed that various parameters of skin resistance reflect changes in the emotional response evoked by snake pictures while heart rate activity increases only when watching pictures of venomous snakes. Various analyses revealed that the respondents’ increased general emotional reactivity, disgust propensity, and specific sensitivity to snake fear measured by psychological questionnaires (ERS, DS-R, and SNAQ) predict the psychophysiological response. High-fear respondents (as compared to low-fear respondents) show a stronger, longer, and more frequent skin resistance response and higher heart rate when watching images of venomous, fear-eliciting snakes. As physiological mechanisms underlying this response may modify the effects of snakebite envenoming, we suggest paying attention not only to the venom itself, but also to the particular species delivering the bite and the victim’s individual sensitivity. It should become an integral part of studies quantifying the effects of envenomation, including studies on animal models.

## Supporting information

S1 Table List of species and picture sources used in the study as visual stimuli. The columns D and E show mean ratings of fear and disgust based on self-reported answers by 112 respondents.
(XLSX)
